# rs622342 in *SLC22A1*, CYP2C9*2 and CYP2C9*3 and Glycemic Response in Individuals with Type 2 Diabetes Mellitus Receiving Metformin/Sulfonylurea Combination Therapy: 6-Month Follow-Up Study

**DOI:** 10.3390/jpm10020053

**Published:** 2020-06-20

**Authors:** Khaled Naja, Ali Salami, Said El Shamieh, Rajaa Fakhoury

**Affiliations:** 1 Department of Biological Sciences, Faculty of Science, Beirut Arab University, Beirut P.O. Box 11-5020, Lebanon; najakhaled@hotmail.com; 2 Rammal Hassan Rammal Research Laboratory, Physio-toxicity (PhyTox) Research Group, Faculty of Sciences (V), Lebanese University, Nabatieh P.O. Box 6573/14, Lebanon; a.salami@ul.edu.lb; 3 Department of Medical Laboratory Technology, Faculty of Health Sciences, Beirut Arab University, Beirut P.O. Box 11-5020, Lebanon

**Keywords:** type 2 diabetes mellitus, single nucleotide polymorphisms, *SLC22A1*, *OCT1*

## Abstract

Background and Objective: Since the treatment outcome with oral anti-diabetics differs between individuals, the objective of this study is to evaluate the significance of rs622342 in *SLC22A1*, CYP2C9*2 (rs1799853) and CYP2C9*3 (rs1057910) with regard to the efficacy of metformin/sulfonylurea combination therapy in individuals with type 2 diabetes mellitus (T2DM). Methods: Eighty-eight Lebanese individuals with T2DM received metformin/sulfonylurea combination therapy over 3 and 6 months. The clinical and biochemical characteristics were collected. Genotyping of rs622342 in *SLC22A1*, CYP2C9*2 and CYP2C9*3 was performed using hybridization probes on real-time polymerase chain reaction (PCR) instrument. Statistical analysis was performed using SPSS 22.0. Results: The levels of fasting blood sugar (FBS) and glycated hemoglobin (HbA1c) showed a statistically significant reduction over 3 and 6 months of follow-up (*p* < 0.001). An interaction between rs622342 in *SLC22A1*, CYP2C9*2 and CYP2C9*3 (*p* = 0.035) was found associated with reduced levels of HbA1c levels after 3 and 6 months. A significant difference between the means of HbA1c was observed among the different groups after 3 and 6 months (*p* = 0.004 and *p* < 0.001, respectively). The most beneficial group was; AA and AC, *1*3, whereas the individuals that benefited the least were CC, *1*3 at 3 and 6 months. In contrast to HbA1c, no interaction was found between the three polymorphisms to affect FBS (*p* = 0.581). Conclusion: The combination of metformin/sulfonylurea therapy led to the maximum glycemic control in individuals with T2DM carrying AA or AC genotypes in *SLC22A1* and *1*3 in *CYP2C9*.

## 1. Introduction

Type 2 diabetes mellitus (T2DM) has reached the level of a global epidemic [1]. Its prevalence is still rising and it is estimated to affect over 400 million people worldwide [2], especially in the developing world and low-income countries [3]. Basic therapeutic interventions in T2DM include diet, physical activity, weight loss and oral anti-diabetic drugs and/or insulin therapy [4]. 

Since monotherapy may fail to achieve its goal in reaching glycemic control in individuals with T2DM [5], combination therapy is usually required and should involve agents with different and complementary mechanisms of action [6]. Recent studies show that early combination therapies were associated with better glycemic control [7]. Metformin pairs well with almost all other anti-diabetic medications [8]. The combination of metformin and sulfonylureas is among the most commonly prescribed [8]. According to a recent consensus report on the management of hyperglycemia in T2D by the American Diabetes Association and European Association for the Study of Diabetes, sulfonylureas are considered a reasonable option for a second-line glucose lowering agent [9], especially when the cost is a major issue [10]. An updated review concluded that the addition of a sulfonylurea to metformin persists the most cost-effective second-line therapy for patients when compared to other drugs (Sodium-glucose co-transporter-2 inhibitors, dipeptidyl-peptidase inhibitors and Glucagon-like peptide-1receptor agonist) [11]. Despite this, it is becoming increasingly evident that the treatment outcome with oral anti-diabetics differs strongly between individuals and that a personalized approach would make sense [12]. 

*Solute carrier family 22 member 1* (*SLC22A1*) encodes an Organic Cation Transporter 1 (OCT1) that is essential for metformin transport into the hepatocytes and subsequent activation [13]. *SLC22A1* is highly polymorphic, and its single nucleotide polymorphisms (SNPs) have been shown to affect the transporter function, causing inter-patient differences in metformin efficacy and disposition [14]. We have recently shown that rs622342 in *SLC22A1* may be associated with variability in therapeutic efficacy of metformin [15]. On the other hand, Cytochrome P450 2C9 (CYP2C9), the hepatic enzyme, is involved in the metabolism of 10%–20% of currently used drugs and has a major role in the metabolism of sulfonylureas [16]. Many SNPs in *CYP2C9* are associated with decreased enzymatic activity [16]; for instance, CYP2C9*2 (rs1799853) and CYP2C9*3 (rs1057910) in exons 3 and 7, respectively, both of which are associated with impaired function and poor metabolism phenotypes [16]. Based on all the above, the objective of this study is to evaluate the significance of rs622342 in *SLC22A1* along with CYP2C9*2 and *3 alleles with regard to the efficacy of metformin/sulfonylurea combination therapy in 88 Lebanese individuals with T2DM followed over a period of 6 months.

## 2. Materials and Methods

### 2.1. Ethics Statement and Patient Recruitment

The institutional review board of Beirut Arab University approved this study (2019A-0043-S-P-0338), and all procedures used in this study were in accordance with the tenets of the Declaration of Helsinki. A detailed history was taken from all participants and a written informed consent was obtained from each patient before participating in the study. 

Eighty-eight unrelated Lebanese individuals (54 men and 34 women) with uncontrolled glycemia were recruited. Uncontrolled glycemia is defined as glycated hemoglobin (HbA1c) ≥ 7.0% according to Glycemic Targets: Standards of Medical Care in Diabetes—2019. All selected patients were started on metformin/glibenclamide 500 mg/5 mg fixed dose combination therapy. Elderly patients; patients with a history of liver or renal disease, congestive cardiac failure, myocardial infarction, cancer, and retinopathy; those treated with insulin during the past 6 months; and those who were pregnant or lactating, were excluded. All selected patients were recommended to take the drug with meals and to drink plenty of water. The included patients completed the study without or with very few mild side effects (mainly mild hypoglycemic events).

### 2.2. Biochemical Measurements

Body mass index (BMI) measurements were collected at the first visit only. Average fasting blood sugar (FBS), HbA1c and fasting lipid profile, total cholesterol (TC), triglycerides (TG), high-density lipoprotein cholesterol (HDL-C), and low-density lipoprotein cholesterol (LDL-C) were recorded as a baseline before treatment, and after three months and six months of treatment.

HbA1c levels were quantified using a D-10 Hemoglobin Testing System (Bio-Rad, Kaki Bukit, Singapore), and the other biochemical parameters were estimated using UniCel DxC 600 Synchron Clinical Systems (Beckman Coulter, Wycombe, UK).

### 2.3. DNA Sampling and Genotyping

Whole blood samples were collected using Ethylenediaminetetraacetic acid (EDTA) tubes. DNA extraction was performed using a GenElute Blood Genomic DNA Kit (Sigma-Aldrich, USA) according to the manufacturer’s manual. The DNA concentration and quality were assessed using a Genova Nano spectrophotometer (Jenway, Staffordshire, UK). SimpleProbe^®^ real-time polymerase chain reaction (PCR) assays for rs622342 in *SLC22A1*, CYP2C9*2 and CYP2C9*3 were performed using a LightSNiP Kit (TIB MOLBIOL GmbH, Berlin, Germany). All the details of the PCR procedure were as previously described [15]. Amplification was performed on a CFX96™ (Bio-Rad, Singapore) real-time thermal cycler, and the data acquisition and melting-curve analyses were performed using CFX Manager™ software (Bio-Rad, Singapore).

### 2.4. Statistical Analysis

The statistical analysis was performed using SPSS (IBM Corp. Released 2013, SPSS Statistics for Windows Version 22.0, Armonk, NY, USA). Categorical and continuous variables were expressed as frequencies and mean ± standard deviation, respectively. Quantitative variables were tested for normality distribution using the Kolmogorov–Smirnov test. The repeated-measures ANOVA was used to show whether there was a statistically significant reduction overtime for the HbA1c test and the FBS test. Then, a post-hoc analysis with a Bonferroni test was used to confirm when the differences occurred, comparing to baseline. The Mann–Whitney test was used to study whether there was a significant difference between the two genotypic groups (AA and AC; CC) over the three moments of testing (at baseline, after 3 and 6 months). The level of significance was set at *p <* 0.05 for all statistical analyses.

## 3. Results

The demographic characteristics of the study participants are shown in Table 1. The group of participants comprised 34 females (38.6%) and 54 males (61.4%). The mean age was 56.6 years old. The clinical characteristics are shown in Table 2. 

**Figure 1:**
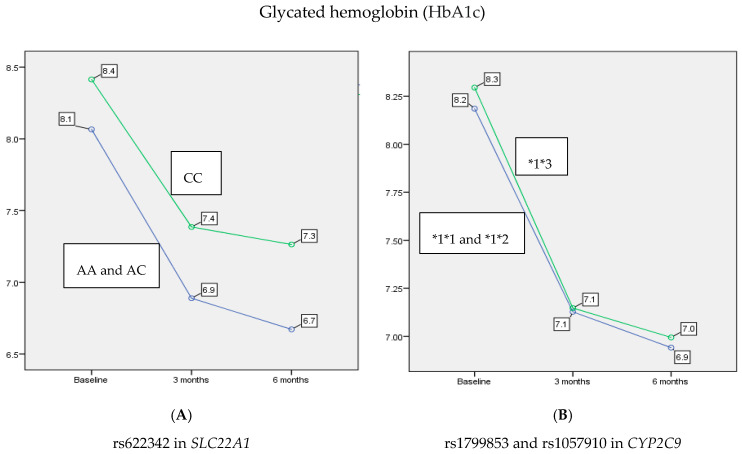
The main effect of rs622342 in *SLC22A1* and rs1799853/rs1057910 in *CYP2C9* on reducing the average of HbA1c after 3 and 6 months. rs622342 in *SLC22A1* was significantly associated with HbA1c levels (*p* < 0.001). rs1799853 and rs1057910 in *CYP2C9* did not show any significant effect on HbA1c levels (*p* = 0.623). HbA1c: glycated hemoglobin; FBS: fasting blood sugar.

The levels of HbA1c showed a statistically significant reduction over the time of testing (*p* < 0.001). Specifically, there was a significant decrease at 3 and 6 months (13.4% and 15.4%, respectively) in the mean of HbA1c comparing to baseline (*p* < 0.001 and *p* < 0.001, respectively). Also, there was a statistically significant decrease of 2.4% in HbA1c between 3 and 6 months (*p* < 0.001). Moreover, this analysis revealed a main effect of rs622342 in *SLC22A1* on HbA1c levels (*p* < 0.001). In contrast, rs1799853 and rs1057910 in *CYP2C9* did not show any significant effect on HbA1c levels when stratified according to *1*1 and *1*2 versus *1*3 (*p* = 0.623). As shown in Figure 1, patients with A allele benefited by about 6.3% of a reduction in HbA1c after 3 months, compared to patients with C allele (AA and AC 6.97 ± 0.41, CC 7.41 ± 0.48, *p* = 0.003), noting that this reduction persisted after 6 months of treatment by about a 7.4% reduction more in patients with A allele compared to those with the C allele (AA and AC 6.76 ± 0.40, CC 7.26 ± 0.44, *p* < 0.001).

An interaction between rs622342 in *SLC22A1* and CYP2C9*2 and CYP2C9*3 (*p* = 0.035) was also associated with reduced levels of HbA1c after 3 and 6 months of treatment with combination therapy. In order to study the interaction between rs622342 in *SLC22A1* and rs1799853/rs1057910 in *CYP2C9*, we examined the reduction in the mean of the HbA1c between four groups: (AA and AC, *1*1 and *1*2), (AA and AC, *1*3), (CC, *1*1 and *1*2) and (CC, *1*3) at baseline, after 3 months and 6 months. At baseline, no significant difference between the means of HbA1c was observed among the four groups (*p* = 0.062). A significant difference between the means of HbA1c was observed among the four groups after 3 and 6 months (*p* = 0.004 and *p* < 0.001, respectively). The most beneficial group was (AA and AC, *1*3) at 3 and 6 months (6.77 ± 0.27 and 6.54 ± 0.19, respectively). Individuals that benefited the least were (CC, *1*3) at 3 and 6 months (7.53 ± 0.51 and 7.45 ± 0.52, respectively).

**Figure 2:**
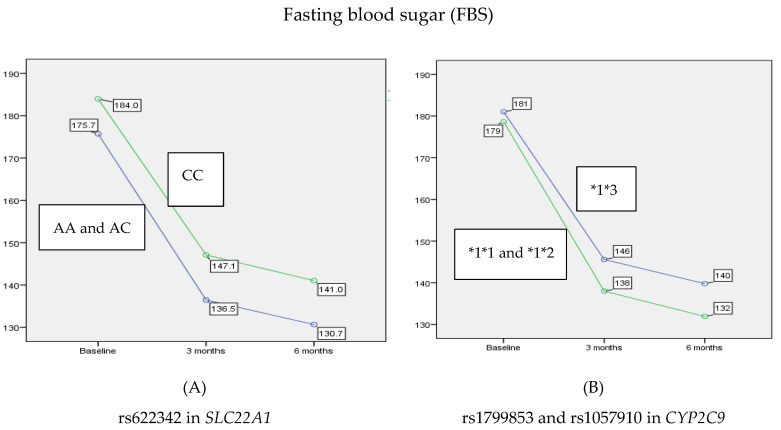
The main effect of rs622342 in *SLC22A1* and rs1799853/rs1057910 in *CYP2C9* on reducing the average of FBS after 3 and 6 months. rs622342 in *SLC22A1* was negatively associated with FBS (*p* = 0.021). rs1799853 and rs1057910 in *CYP2C9* did not show any significant effect on FBS levels (*p* = 0.156). HbA1c: glycated hemoglobin; FBS: fasting blood sugar.

Similar to HbA1c, the FBS levels showed a statistically significant reduction over time (*p* < 0.001). When a post-hoc analysis was performed, it showed that at 3 and 6 months there was a statistically significant decrease (21.2%, *p* < 0.001 and 24.4%, *p* < 0.001, respectively) in the mean of FBS compared to the baseline. Likewise, there was a statistically significant decrease of 4.2% in FBS between 3 and 6 months (*p* < 0.001). Moreover, this analysis revealed a major effect of rs622342 in *SLC22A1* on reducing the average of FBS over 6 months (*p* = 0.021). In contrast, rs1799853 and rs1057910 in *CYP2C9* did not show any significant effect on FBS levels when stratified according to 1*1 and *1*2 versus *1*3 (*p* = 0.156). Similarly, no interaction was found between rs622342 in *SLC22A1* and rs1799853/rs1057910 in *CYP2C9* (*p* = 0.581) in reducing the average of FBS after 3 and 6 months of treatment with combination therapy. As shown in Figure 2, patients with A allele were able to benefit more with 7.7% of reduction in FBS after 3 months compared to patients with C allele (AA and AC 136.58 ± 14.66, CC 147.14 ± 16.11, *p* = 0.004), and this reduction remained after 6 months of treatment by about 7.9% of reduction more in patients with A allele comparing to those with C allele (AA and AC 130.72 ± 14.64, CC 141.01 ± 15.79, *p* = 0.008).

All lipid levels decreased over 3 and 6 months of combinational therapy (Table 2), yet neither an association nor an interaction between rs622342 in *SLC22A1*, CYP2C9*2 and CYP2C9*3 were detected over any of the lipid levels (*p* > 0.05).

## 4. Discussion

The results show that the individuals carrying rs622342AA and rs622342AC genotypes in *SLC22A1* and those having *1*3 genotype in *CYP2C9* experienced the highest HbA1c reduction after metformin/sulfonylurea combination therapy when compared to other groups (individuals with rs622342CC and *1*2 genotype in *CYP2C9*).

Metformin and sulfonylureas have complementary mechanisms: while metformin increases the sensitivity to insulin [17], sulfonylureas promote insulin secretion from the pancreatic β cells [18]. The effect of rs622342A>C in *SLC22A1* on metformin action has been widely studied. Supporting the current results, we showed in our previous study that rs622342 in *SLC22A1* may alter metformin pharmacokinetics and consequently its efficacy [15]. This is in the same direction with Becker et al., who showed that in the Rotterdam study the only SNP with significant differences in metformin response was rs622342 *SLC22A1* [19]. In addition, Umamaheswaran et al., who studied rs622342 on Indian population, showed that AA patients had a 5.6 times better chance to respond to metformin treatment when compared to CC patients [20]. Similarly, Reséndiz et al., who studied rs622342 on Mexican population, showed that CC genotype is significantly associated with an increase in HbA1c levels during the follow-up period [21], and Ebid et al. showed in an Egyptian population that carriers of the A allele of rs622342 were more responsive to metformin in combination therapy than C allele carriers [22].

The effect of CYP2C9*2 and CYP2C9*3 on sulfonylureas action remains controversial. Zhou et al. showed that in the GoDARTS study patients with *CYP2C9* *2*2, *2*3 or *2*3 displayed greater odds of achieving the target HbA1c of less than 7% after 18 months of sulfonylureas initiation in combination with metformin [23]. Surendiran et al. showed, in a study conducted on an Indian population, that *CYP2C9* *1*3 and *1*2 were better in responding to treatment with glibenclamide than those with the *1*1 genotype [24]. Castelan et al. showed, in a study conducted on a Mexican population treated with glibenclamide, that CYP2C9*3 allele was associated with good glycemic control, while *CYP2C9* *2 was not [25], noting that Rodriguez et al. showed that HbA1c levels were more regulated among *3 heterozygous individuals in a Mexican population [26]. In contrast, Klen et al. showed, in a study conducted on a Caucasian population, no significant differences in HbA1c reduction among the different genotypes of *CYP2C9* [27]. Likewise, Chen et al. showed that carriers of CYP2C9*2 and *3 alleles may experience an increased insulin response to glipizide, but no effect of genotype was demonstrated in glucose based measurements [28]. Based on our results, we can hypothesize that individuals carrying *1*3 genotype may have higher sulfonylurea plasma level, since they have lower enzyme activity than the wild allele, and consequently higher sulfonylurea levels that enter the beta cells.

Our results showed an interaction between rs622342 in *SLC22A1* and rs1799853/rs1057910 in *CYP2C9* to reduce the average of HbA1c after 3 and 6 months. When these findings are combined with the fact that metformin and sulfonylureas have complementary action, our results may indicate that the beneficial effect of each SNP may be additive. In contrast, no significant difference was noticed on lipid levels among the studied groups.

A limitation to our study is the absence of the homozygous variants in *CYP2C9* (*2*2 and *3*3) which are needed to better assess the interaction effect; this is due to the limited sample size. In contrast, the main strength is that our study is the first to examine the effect of three SNPs in *SLC22A1* and *CYP2C9* genes on the efficacy of the combination therapy.

## 5. Conclusions and Perspectives

In conclusion, the combination of metformin/sulfonylurea therapy led to the maximum glycemic control in individuals with T2DM carrying AA or AC genotypes in *SLC22A1* and *1*3 in *CYP2C9.* Our results might enable the achievement of optimal glucose control and the improvement of therapeutic efficacy, and might be beneficial for the future tailoring of personalized therapy in patients with T2DM. Future studies of such associations in larger sample sizes are warranted to reveal significant insights into the clinical application. 

## Figures and Tables

**Table 1:** Demographic and genetic characteristics of the study participants.

Variables	Study Participants (*n* = 88)
Age	56.6 ± 5
Gender *n* (%)	
Male	54 (61.4)
Female	34 (38.6)
BMI (Kg/m^2^)	25.13 ± 1.35
MAF	
rs622342 in *SLC22A1*	0.41
Genotypes	
AA *n* (%)	33 (37.5)
AC *n* (%)	38 (43.2)
CC *n* (%)	17 (19.3)
rs1799853/rs1057910 in *CYP2C9*	0.31
Genotypes	
*1*1 *n* (%)	51 (58.0)
*1*2 *n* (%)	20 (22.7)
*1*3 *n* (%)	17 (19.3)

Values are arithmetic mean ± SD for continuous variables. Categorical variables are shown as number (*n*) and percentages (%). *n*: sample size. BMI: body mass index; MAF: minor allele frequency; *SLC22A1*: Solute carrier family 22 member 1; *CYP2C9:* Cytochrome P450 Family 2 Subfamily C Member.

**Table 2:** Clinical characteristics of the study participants.

Variables	Baseline	After 3 Months	After 6 Months	*p*
HbA1c (mmol/mol)	8.1 ± 0.4	7.03 ± 0.44	6.84 ± 0.4	<0.001
FBS (mg/dL)	177.6 ± 12.7	140.2 ± 15.6	134.7 ± 15.4	<0.001
Total cholesterol (mg/dL)	215.3 ± 16.6	208.9 ± 14.7	203.7 ± 15.4	<0.001
LDL-C (mg/dL)	135.3 ± 18.3	128.6 ± 16.3	120.1 ± 17.5	<0.001
HDL-C (mg/dL)	42.7 ± 5.3	45.7 ± 4.9	47.7 ± 5.18	<0.001
Triglycerides (mg/dL)	175.4 ± 15.5	165.1 ± 14.2	161.7 ± 13.83	<0.001

Values are arithmetic mean ± SD for continuous variables. LDL-C: low-density lipoprotein cholesterol; HDL-C: high-density lipoprotein cholesterol; HbA1c: glycated hemoglobin; FBS: fasting blood sugar.
